# Supplementary material for: In Vitro Fermentation Shows Polyphenol and Fiber Blends Have an Additive Beneficial Effect on Gut Microbiota States
Source: Nutrients. 2024 Apr 13;16(8):1159. doi: 10.3390/nu16081159 (PMC11053737; doi:10.3390/nu16081159)
Supplement: Supplementary file 1 [file nutrients-16-01159-s001.zip › Supplementary Figures_revised/Whitman et al_ polyphenol fiber supplementation_supplementary figure_final_04122024.pdf]

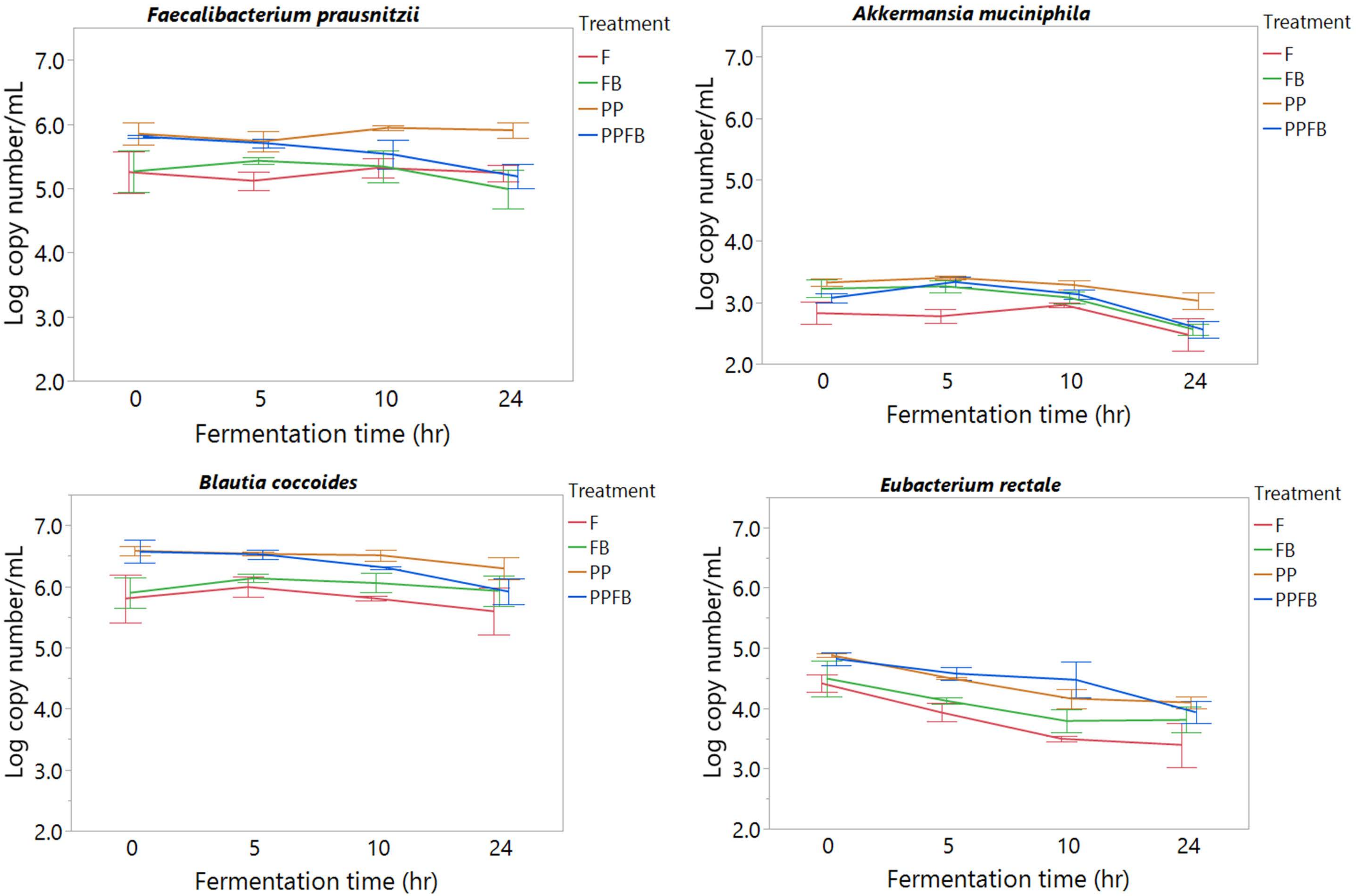

Figure S1: Compositional analysis of selected bacterial taxa and abundance changes over time due to supplementation. *Faecalibacterium prausnitzii* (A), *Akkermansia muciniphila* (B), *Blautia coccoides* (C), *Eubacterium rectale* (D). As fermentation residence time increases, differential changes occur within the community, but statistically significant differences were not evident ($p \geq 0.1$). F = non-supplemented; PP= polyphenol blend; FB = fiber blend, PPFB – PP and FB blend. Data are Mean ± SEM ($n = 3$)

Table S1. Constituents in polyphenol and fiber blends and solubilization form

Constituent	Active ingredient*	Dry weight	Form
Polyphenols			
Cranberry	0.5 g	880 mg	Powder solubilized in methanol
Blueberry	0.5 g	12600 mg	
Green Tea	0.5 g	500 mg	
Cocoa	0.5 g	1200 mg	
Fiber			
Resistant Starch	20 g	35.8 g	Powder in medium suspension (CCM)
Galacto-oligosaccharides	5 g	6.5 g	
Inulin	5 g	5.5 g	

*Amounts of active ingredients in polyphenol and fiber constituents.

Recommended daily consumption for normal western habitual diet: 2 g/day polyphenols, 30 g/day fiber

Table S2. Bacterial taxa primer sequences for qPCR [17, 26]

Target	Primer	Sequence	Annealing temp (^oC)
Bifidobacterium spp.	BIF164f	GGGTGGTAATGCCGGATG	55
	BIF662r	CCS=ACCGTTACACCGGGAA	
Lactabacillus spp.	SGLAB 0159f	GGAAACAG(A/G)TGCTAATACCG	55
	SGLAB 0667r	CACCGCTACACATGGAG	
Ruminococcus bromii	Bromii F	CGAACGGAACTGTTTTGAAAGA	62
	Bromii R	CAAAACCATGTGGTTCCGATAT	
Dorea spp.	DoreaF	GCAGCTAACGCAATAAGCAG	60
	DoreaR	CATTACGAAGCGGTCATCG	
Faecalbacterium prausnitzii	Fprau 07	CCATGAATTGCCTTCAAAACTGTT	55
	Fprau02	GAGCCTCAGCGTCAGTTGGT	
Akkermansia muciniphila	AM1	CAGCACGTGAAGGTGGGGAC	50
	AM2	CCTTGCGGTTGGCTTCAGAT	
Blautia coccoides	Ccoc-F	AAATGACGGTACCTGACTAA	50
	Ccoc-r	CTTTGAGTTTCATTCTTGCGAA	
Eubacterium rectale	REC-F	CATTGCTTCTCGGTGCCGTC	53.7
	REC-R	ATTTGCTCGGCTTCACAGCT	

Table S3. Polyphenol and fiber blend supplementation kinetic influence on the abundance of select bacterial taxa - pairwise comparison by treatment across time

Bifidobacterium spp.	**0hr**	**5hr**	**10hr**	**24hr**
FB-F	0.9998	**0.0265**	**0.0055**	**0.0059**
PP-F	0.5369	**0.0016**	**0.0092**	0.1711
PPFB-F	0.8592	**0.0029**	**0.0040**	**0.0207**
PP-FB	0.4939	0.1844	0.9750	0.1394
PPFB-FB	0.8221	0.3708	0.9935	0.7755
PPFB-PP	0.9886	0.9459	0.9056	0.4724
Lactobacillus spp.	**0hr**	**5hr**	**10hr**	**24hr**
FB-F	0.9281	0.8385	**0.0018**	**0.0003**
PP-F	0.7514	0.2523	**0.0222**	**0.0074**
PPFB-F	0.5347	**0.0315**	**0.0008**	**0.0002**
PP-FB	0.9767	0.6415	0.2535	0.0847
PPFB-FB	0.8557	0.1012	0.8756	0.9724
PPFB-PP	0.9782	0.4807	0.0910	**0.0464**
Dorea spp.	**0hr**	**5hr**	**10hr**	**24hr**
FB-F	0.9992	0.6002	0.6868	0.4630
PP-F	0.5332	0.0955	**0.0343**	**0.0053**
PPFB-F	0.3151	**0.0096**	**0.0174**	**0.0055**
PP-FB	0.6039	0.4976	0.1658	**0.0400**
PPFB-FB	0.3686	0.0545	0.0827	**0.0418**
PPFB-PP	0.9654	0.3943	0.9575	1.0000
Ruminococcus_bromii	**0hr**	**5hr**	**10hr**	**24hr**
FB-F	0.9977	0.7381	**0.0013**	**0.0320**
PP-F	0.0580	0.5463	0.2300	0.8157
PPFB-F	0.5297	0.3305	**0.0012**	**0.0347**
PP-FB	0.0749	0.9848	**0.0169**	0.1100
PPFB-FB	0.6296	0.8558	0.9998	0.9999
PPFB-PP	0.3872	0.9684	**0.0153**	0.1195

F = non-supplemented; PP = polyphenol blend; FB = fiber blend; PPFB = polyphenol and fiber blend. Bold represents significant differences across treatment groups ($p \leq 0.05$).

Table S4. Differences in total short-chain fatty acid (SCFA) concentrations due to polyphenol and fiber blend supplementation - pairwise comparison by treatment across time

Treatment	Time (hr)	Total SCFA (mM)	P-value Difference to F*	P-value Difference to PPFB**	Ac:Pp:Bu Ratio
**PPFB**	0	0.79	0.801	--	51:29:20
	5	3.59	0.981	--	67:25:08
	10	11.77	0.013*	--	74:14:12
	24	20.18	0.007*	--	57:17:26
**PP**	0	0.89	0.972	0.567	54:28:18
	5	4.11	0.427	0.626	71:21:08
	10	9.75	0.244	0.213	59:17:24
	24	14.93	0.136	0.126	60:16:24
**FB**	0	0.83	0.984	0.941	54:28:18
	5	3.27	0.972	0.851	68:24:08
	10	11.54	0.018*	0.995	80:12:08
	24	19.13	0.0157*	0.847	53:18:29

F = non-supplemented; PP = polyphenol blend; FB = fiber blend; PPFB = polyphenol and fiber blend. Ratio of acetic acid, propionic acid, and butyric acid (Ac:Pp:Bu) in samples. *Significant differences across treatment compared to F at the same time point ($p \leq 0.05$); **Significant differences across treatment compared to PPFB at the same time point ($p \leq 0.05$). Mean±SEM values ($n = 3$)

Table S5. Mean short-chain fatty acid (SCFA) concentrations due to polyphenol and fiber blend supplementation

Treatment	Time (hr)	Acetic Acid	Propionic Acid	Isobutyric Acid	Butyric Acid	Isovaleric Acid	Valeric Acid
PPFB	0	0.40 ± 0.08	0.23 ± 0.01	0.01 ± 0.00	0.16 ± 0.01	0.02 ± 0.00	0.02 ± 0.00
	5	2.41 ± 0.54	0.88 ± 0.11	0.02 ± 0.00	0.30 ± 0.05	0.03 ± 0.00	0.03 ± 0.00
	10	8.72 ± 1.21	1.64 ± 0.37	0.01 ± 0.00	1.41 ± 0.62	0.03 ± 0.00	0.03 ± 0.01
	24	11.54 ± 2.75	3.36 ± 0.76	0.06 ± 0.05	5.29 ± 2.14	0.21 ± 0.17	0.60 ± 0.47
PP	0	0.48 ± 0.08	0.25 ± 0.03	0.01 ± 0.00	0.16 ± 0.02	0.02 ± 0.00	0.02 ± 0.00
	5	2.91 ± 0.30	0.88 ± 0.08	0.02 ± 0.00	0.32 ± 0.03	0.03 ± 0.00	0.02 ± 0.00
	10	5.77 ± 0.33	1.67 ± 0.06	0.02 ± 0.00	2.31 ± 0.24	0.04 ± 0.00	0.06 ± 0.01
	24	8.89 ± 0.34	2.37 ± 0.89	0.19 ± 0.03	3.67 ± 1.26	0.58 ± 0.10	1.32 ± 0.21
FB	0	0.45 ± 0.06	0.23 ± 0.02	0.01 ± 0.00	0.15 ± 0.01	0.02 ± 0.00	0.02 ± 0.00
	5	2.21 ± 0.07	0.79 ± 0.04	0.02 ± 0.00	0.27 ± 0.02	0.03 ± 0.00	0.02 ± 0.00
	10	9.22 ± 2.13	1.40 ± 0.15	0.02 ± 0.00	0.92 ± 0.34	0.03 ± 0.00	0.03 ± 0.00
	24	10.22 ± 3.22	3.34 ± 0.90	0.05 ± 0.04	5.57 ± 3.03	0.22 ± 0.18	0.63 ± 0.51
F	0	0.45 ± 0.02	0.24 ± 0.01	0.01 ± 0.00	0.16 ± 0.00	0.02 ± 0.00	0.02 ± 0.00
	5	2.33 ± 0.45	0.84 ± 0.13	0.02 ± 0.00	0.26 ± 0.07	0.03 ± 0.00	0.02 ± 0.00
	10	5.18 ± 0.60	1.60 ± 0.25	0.02 ± 0.00	1.03 ± 0.19	0.04 ± 0.00	0.05 ± 0.01
	24	6.97 ± 2.59	2.11 ± 1.33	0.16 ± 0.14	2.62 ± 1.48	0.44 ± 0.29	1.33 ± 1.18

F = non-supplemented PP = polyphenol blend; FB = fiber blend; PPFB = polyphenol and fiber blend. All values: Mean(mM)±SEM (n = 3)

Table S6. Mean concentrations of select microbial metabolites due to polyphenol and fiber blend supplementation

Treatment	Time (hr)	Indole (uM)±SEM	Ammonia (mM)±SEM	FRAP (mM)±SEM
PPFB	0	37.50 ± 6.68	6.24 ± 6.71	8.047 ± 0.549
	5	29.31 ± 10.19	7.89 ± 6.92	8.788 ± 0.322
	10	17.64 ± 0.55	9.07 ± 7.57	8.075 ± 0.34
	24	-4.93 ± 1.31	4.27 ± 3.27	5.642 ± 0.328
PP	0	53.34 ± 9.6	5.72 ± 6.94	7.642 ± 0.659
	5	34.13 ± 4.56	6.88 ± 9.85	8.596 ± 0.74
	10	29.83 ± 1.43	9.95 ± 11.11	8.279 ± 0.343
	24	29.58 ± 0.36	9.48 ± 1.62	5.626 ± 0.163
FB	0	55.53 ± 1.48	5.68 ± 6.46	3.995 ± 0.184
	5	32.79 ± 6.29	7.54 ± 8.98	4.436 ± 0.487
	10	18.22 ± 6.85	8.93 ± 10.23	4.593 ± 0.386
	24	7.14 ± 3.52	6.74 ± 2.14	2.799 ± 0.502
F	0	31.37 ± 6.31	9.26 ± 7.93	3.925 ± 0.204
	5	31.37 ± 6.31	11.41 ± 9.67	4.163 ± 0.26
	10	22.60 ± 3.43	9.34 ± 5.10	4.486 ± 0.305
	24	46.39 ± 0.71	21.02 ± 2.17	3.275 ± 0.136

F = non-supplemented, PP = polyphenol blend; FB = fiber blend; PPFB = polyphenol and fiber blend. Values represent Mean±SEM (n = 3)

Table S7. Select metabolite pairwise comparison across time by treatment due to polyphenol and fiber blend supplementation

Treatment	Time 1 (hr)	Time 2 (hr)	Indole p-value	Ammonia p-value	FRAP p-value
PPFB	0	5	0.337	0.14	0.337
	0	10	0.182	0.948	1.000
	0	24	**0.0004***	1.000	0.193
	5	10	1.000	1.000	0.66
	5	24	**0.036***	1.000	0.078
	10	24	0.178	1.000	**0.043**
PP	0	5	0.133	1.000	0.738
	0	10	0.227	1.000	1.000
	0	24	0.281	1.000	0.305
	5	10	0.84	0.864	1.000
	5	24	1.000	1.000	0.147
	10	24	1.000	1.000	**0.012**
FB	0	5	0.221	1.000	1.000
	0	10	0.097	1.000	0.586
	0	24	**0.021***	1.000	0.175
	5	10	**0.003***	1.000	1.000
	5	24	**0.023***	1.000	0.245
	10	24	0.172	1.000	0.263
F	0	5	1.000	1.000	1.000
	0	10	1.000	1.000	1.000
	0	24	0.328	0.738	**0.042**
	5	10	1.000	1.000	0.362
	5	24	0.328	1.000	0.362
	10	24	**0.022***	0.405	0.232

F = non-supplemented; PP = polyphenol blend; FB = fiber blend; PPFB = polyphenol and fiber blend. *Significant differences across time within each treatment ($p<0.05$).
