# Supplementary figures and images for: In Vitro Fermentation Shows Polyphenol and Fiber Blends Have an Additive Beneficial Effect on Gut Microbiota States
Source: Nutrients. 2024 Apr 13;16(8):1159. doi: 10.3390/nu16081159 (PMC11053737; doi:10.3390/nu16081159)

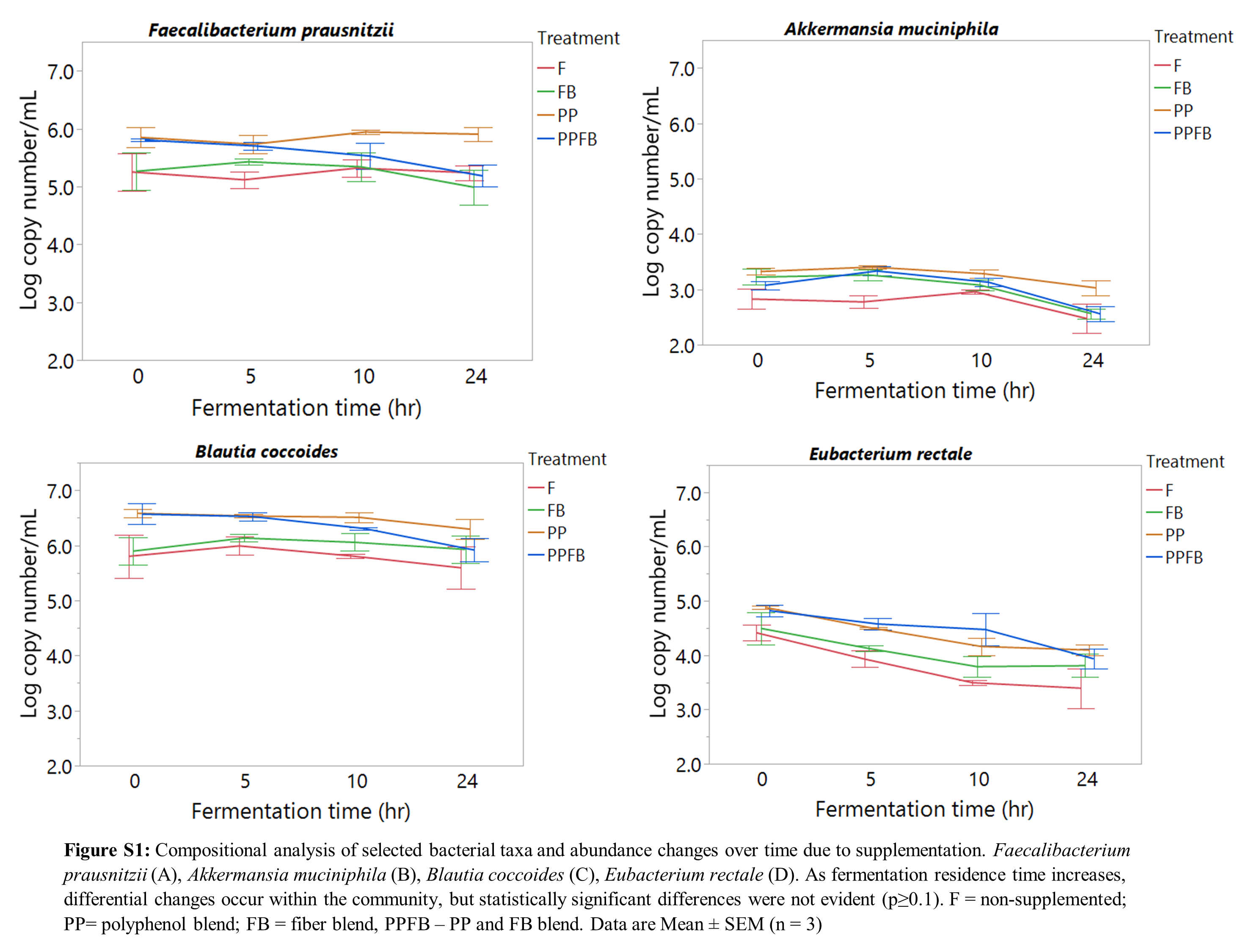

Supplement: Supplementary file 1 [file nutrients-16-01159-s001.zip › Supplementary Figures_revised/Figure S1_revised.tif]

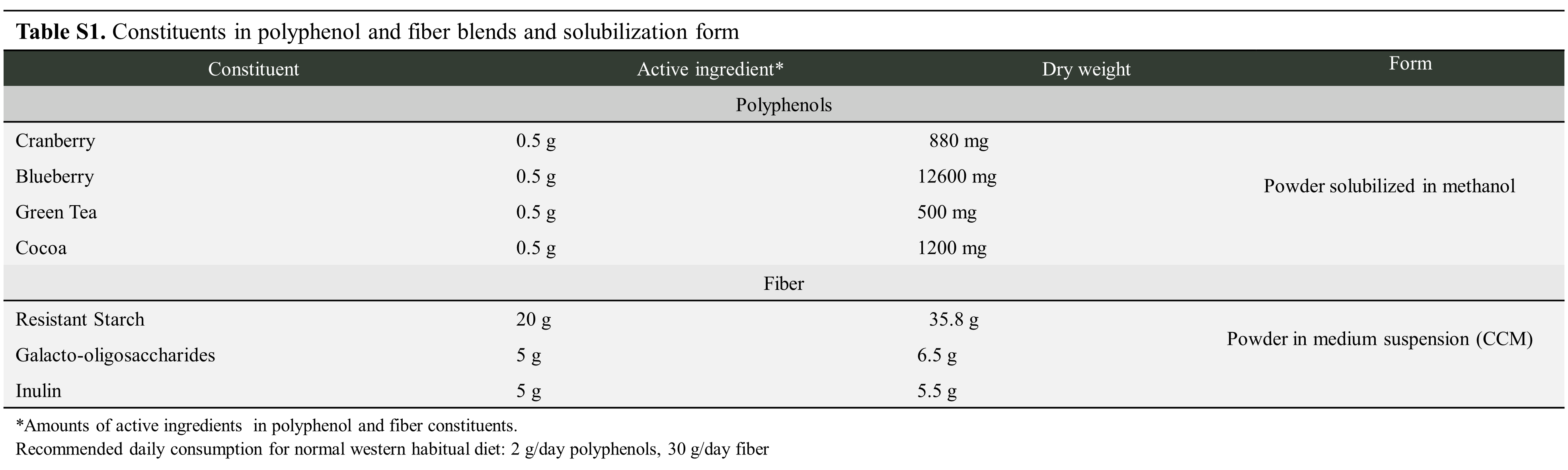

Supplement: Supplementary file 1 [file nutrients-16-01159-s001.zip › Supplementary Figures_revised/Table S1.tif]

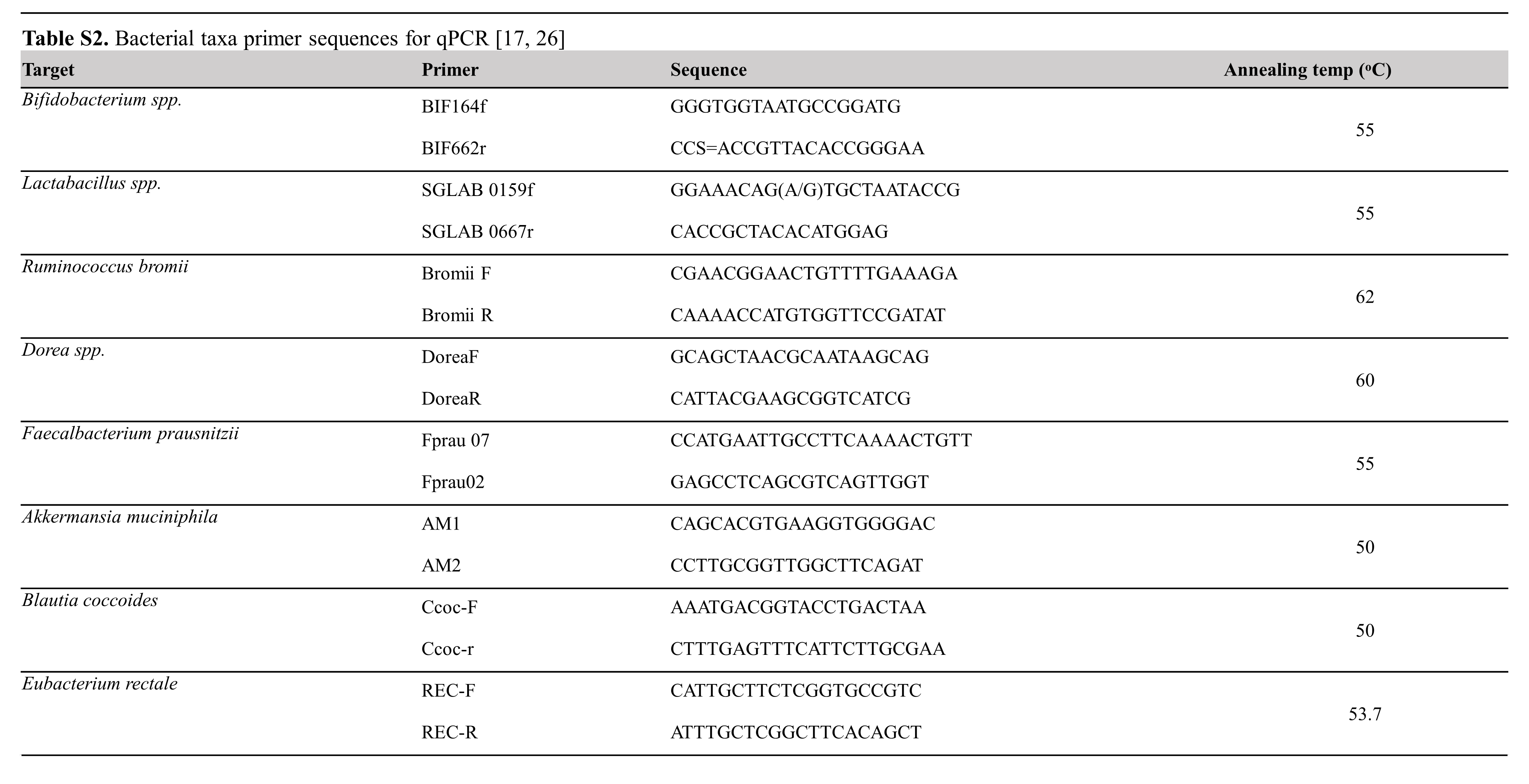

Supplement: Supplementary file 1 [file nutrients-16-01159-s001.zip › Supplementary Figures_revised/Table S2_revised.tif]

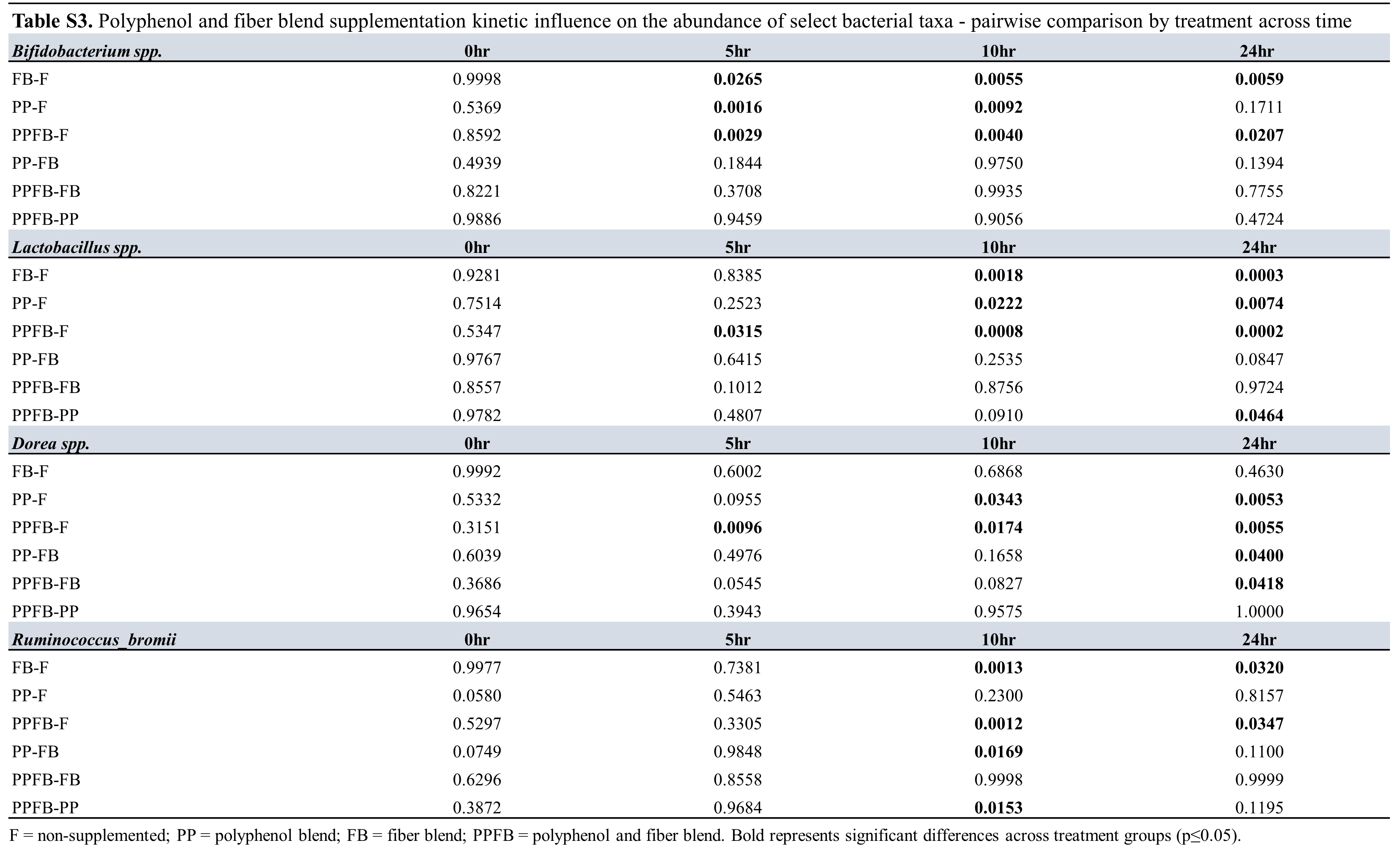

Supplement: Supplementary file 1 [file nutrients-16-01159-s001.zip › Supplementary Figures_revised/Table S3.tif]

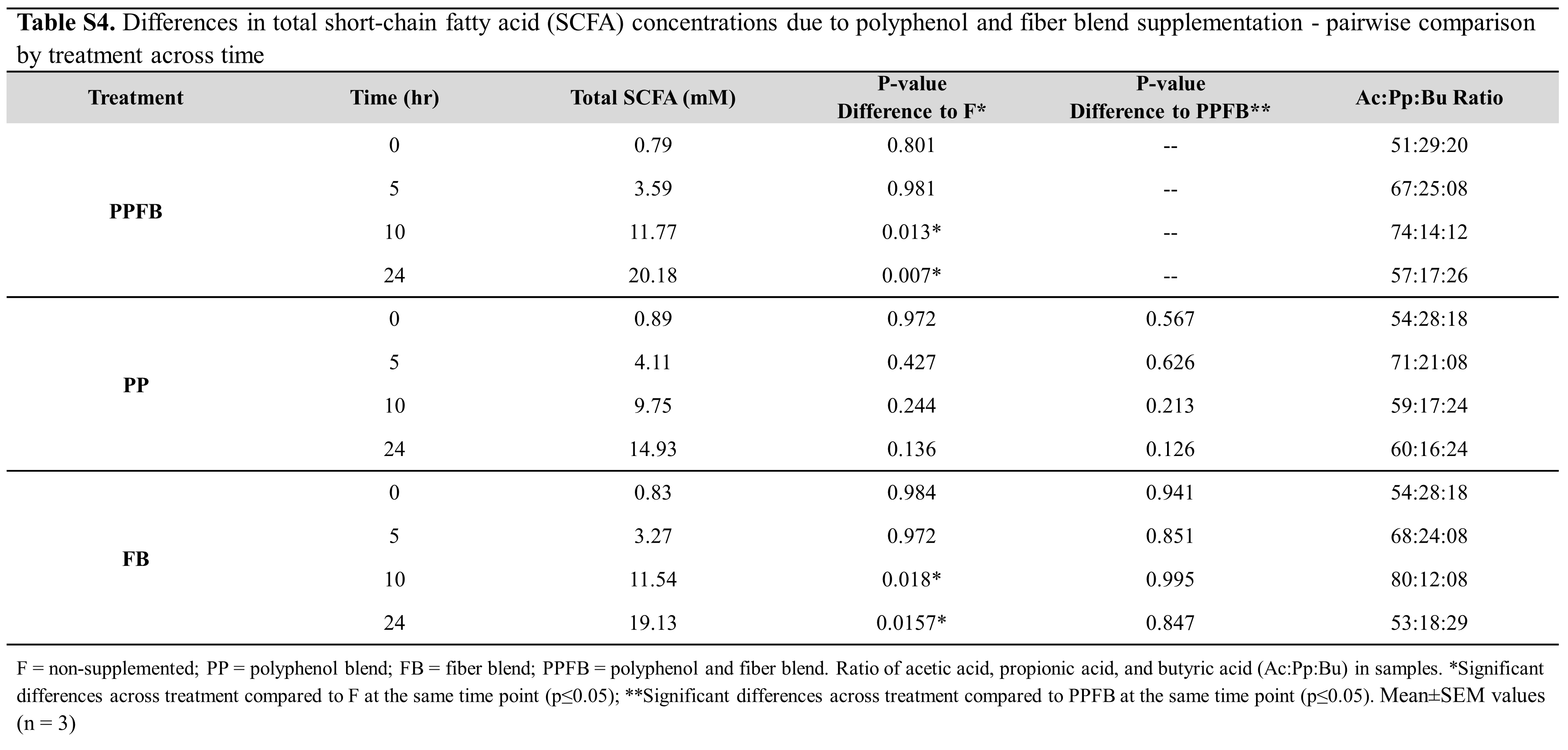

Supplement: Supplementary file 1 [file nutrients-16-01159-s001.zip › Supplementary Figures_revised/Table S4.tif]

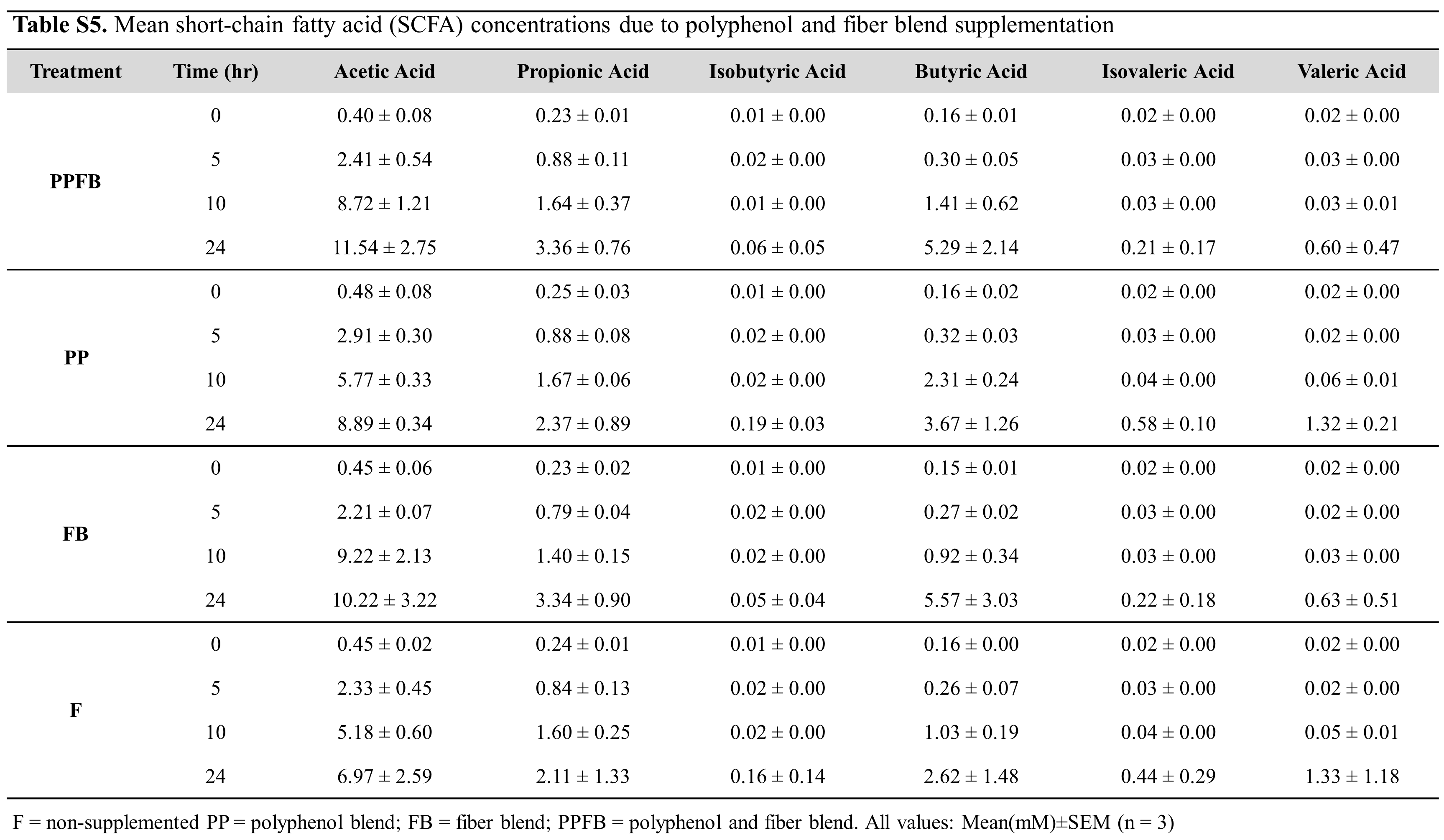

Supplement: Supplementary file 1 [file nutrients-16-01159-s001.zip › Supplementary Figures_revised/Table S5.tif]

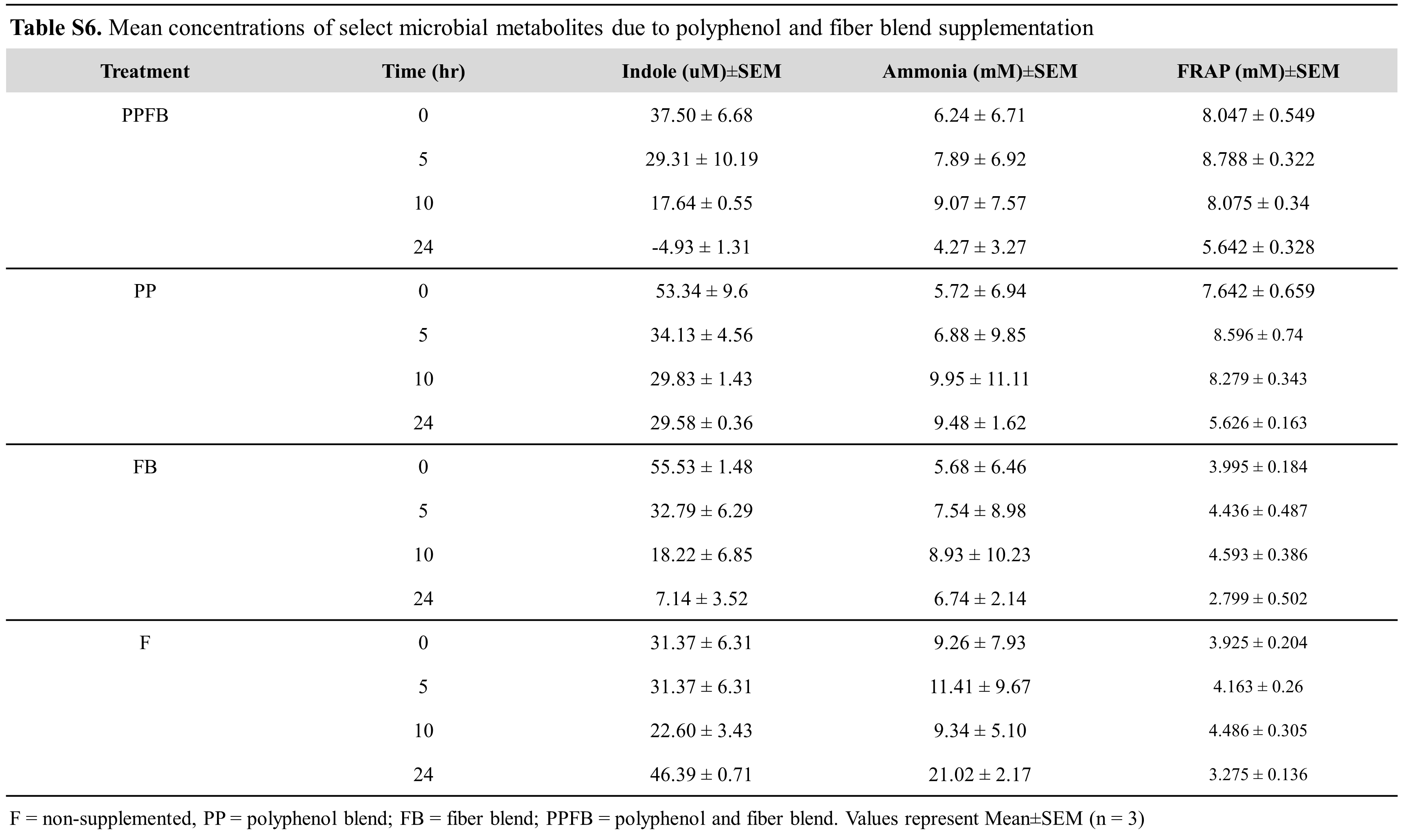

Supplement: Supplementary file 1 [file nutrients-16-01159-s001.zip › Supplementary Figures_revised/Table S6.tif]

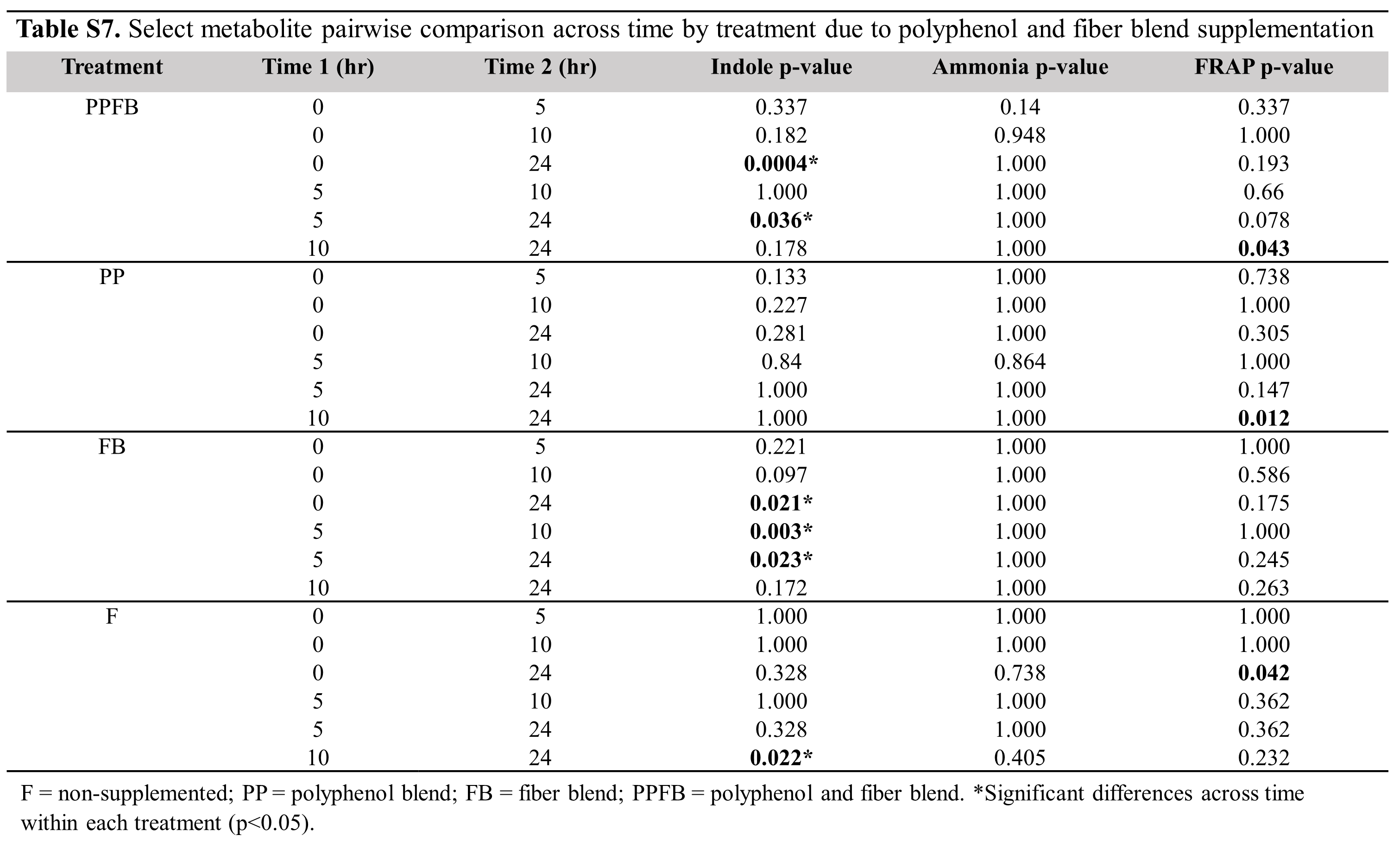

Supplement: Supplementary file 1 [file nutrients-16-01159-s001.zip › Supplementary Figures_revised/Table S7.tif]
